# 849 Rapid Vascularized Native Collagen/elastin Matrix Offers Multiple New Surgical Options in Burns and Trauma

**DOI:** 10.1093/jbcr/iraf019.380

**Published:** 2025-04-01

**Authors:** Markus Oehlbauer

**Affiliations:** Burn Center BG Trauma Center Murnau

## Abstract

**Introduction:**

Despite successful defect coverage by means of complex skin or muscle flaps, particularly large and deep problematic wounds with exposed bradytrophic tissues after soft tissue loss are highly susceptible to surgical revision. This has led to the development of dermal matrices in order to improve quality and function of reconstructed tissue.

Native dermal tissue matrix, consisting of native collagen (collagen type I, III and V) supplemented by an elastin hydolyzate was first used in 1mm thickness for the treatment in one-stage procedures and in 2mm thickness for the treatment in two-stage procedures. Due to the rapid vascularisation of this collagen/elastin matrix more and more former 2 mm-two-stage procedures showed to able to be performed one-stage.

**Methods:**

93 patients treated in a level I trauma center between January 2014 and December 2020 in terms of severe soft tissue defects of upper or lower extremity using STSG in combination with native collagen-elastin tissue matrix in one or two stage procedures were included in a retrospective study. The healing of the soft tissue defect was measured by assessment of the take rate. Outcome quality of the scar tissue was assessed using the Vancouver Scar Scale more than 24 months postoperatively.

**Results:**

Overall healing rate (number of patients with take rate ≥ 75%) was 84%. The majority of postoperative events that had to be revised surgically included healing disturbances such as remaining defects, necrosis, or delayed healing

Regarding the VSS there was no overall difference to treatment groups after split skin grafting to exposed (and/or partial loss of) subcutaneous tissue.

Two-years follow up of these collagen-elastin matrix procedures in defect coverage showed an excellent functional outcome: Up until now, no areas with unstable scars have occurred, no surgical scar revisions were required. The patients were still able to wear normal footwear, clinical gait analysis showed perfect functional outcome.

**Conclusions:**

The application of native collagen-elastin tissue matrix in patients with exposed bradytrophic tissue after severe burn or traumatic injuries treated so far can be recommemded as an excellent reconstruction method, independent of one or two stage procedures.

**Applicability of Research to Practice:**

Use of native collagen-elastin tissue matrix in severe burn and traumatic injuries with exposed bradytrophic tissue can successfully avoid complex skin or muscle flaps and minimize the risk of major surgical revision.

**Funding for the Study:**

N/A

